# Taxation of sugar-sweetened beverages: Perspectives of adults and nursing practitioners in Makhanda, Eastern Cape, South Africa

**DOI:** 10.1177/22799036261455674

**Published:** 2026-09-15

**Authors:** Lanine Herman, Nelene Koen

**Affiliations:** 1 Division of Human Nutrition, Faculty of Medicine and Health Sciences, 26697 Stellenbosch University, Cape Town, South Africa

**Keywords:** sugar-sweetened beverages, health promotion levy, South Africa, taxation, perspectives

## Abstract

**Background:**

High sugar-sweetened beverage (SSB) consumption has been linked to the rising prevalence of obesity and non-communicable diseases (NCDs) in South Africa. In April 2018, the country introduced an SSB tax—known as the Health Promotion Levy (HPL)—as a fiscal measure to reduce excessive sugar consumption and curb the rise of overweight, obesity, and NCDs. Although national evidence indicates product reformulation, community-level awareness and perceptions, particularly in low-socioeconomic settings, remain unclear.

**Objectives:**

To assess adults’ awareness, beliefs, and self-reported behaviour changes related to the SSB tax, and to explore nursing practitioners’ understanding, perceptions of its impact on consumer behaviour, and views on their educational role at primary health care (PHC) facilities in Makhanda, Eastern Cape.

**Design and methods:**

A multi-method, cross-sectional design was used. Quantitative data were collected from adults (n = 275) at seven PHC facilities through an interviewer-administered questionnaire. Qualitative data were obtained from semi-structured interviews with nursing practitioners (n = 10) at the same facilities. Data were analysed using descriptive statistics and thematic analysis, and the results were integrated for interpretation.

**Results:**

Ninety-six percent of adults (n = 264), based on the interviewer-administered questionnaire, were unaware of the SSB tax, while 98% (n = 269) reported no change in consumption since implementation. Nursing practitioners displayed limited awareness of the tax’s rationale and timing but supported taxation as a public health strategy.

**Conclusions:**

The SSB tax demonstrates limited perceived impact in this setting. Strengthened community education, awareness campaigns, and improved policy implementation are required to enhance effectiveness.

## Significance for public health

This study provides critical insights into how fiscal measures, such as the SSB tax, are perceived and understood within low socioeconomic status communities in SA. The finding that most adults were unaware of the SSB tax and that nursing practitioners themselves lacked clarity about its rationale highlights a substantial gap in health communication. Fiscal policies alone are insufficient to achieve meaningful dietary change without concurrent public education and multi-sectoral collaboration. Nursing practitioners, positioned at the frontline of PHC, can play an influential role in promoting awareness of the harms of SSB consumption and the purpose of taxation measures. However, their effectiveness depends on adequate training, resources, and community-engaging public health campaigns. Strengthening both health education and policy enforcement is essential to enhance the equity and impact of SSB taxation, particularly in resource-constrained settings such as Makhanda.

## Introduction

The global prevalence of non-communicable diseases (NCDs), including obesity and diabetes, constitutes a significant public health challenge, posing substantial threats to social and economic development worldwide.^1^ NCDs are the leading cause of mortality worldwide and, although historically considered a concern of high-income countries, they are increasingly impacting the economies and health systems of low- and middle-income countries.^2^ In sub-Saharan Africa (SSA), including South Africa (SA), the burden of nutrition-related NCDs is rising. A major contributing factor is the high and growing prevalence of overweight and obesity, which in turn accelerates the incidence of nutrition-related NCDs. Many of these premature deaths are preventable through lifestyle modifications, particularly the reduction of free sugar intake.^2^

Fiscal policies, such as sugar-sweetened beverage (SSB) taxation, have emerged as an evidence-based strategy to discourage consumption by increasing the retail price of these beverages. International experiences provide compelling evidence for the effectiveness of SSB taxes. Countries such as Mexico,^3^ the United Kingdom (UK)^,4^ and SA have implemented taxes on SSBs with varying levels of success, reporting reductions in purchasing and consumption, particularly among low- and middle-income populations. These examples underscore the potential of taxation as a population-level intervention to improve dietary behaviour and reduce the burden of NCDs.^2^

In Mexico, the introduction of a 10% SSB tax in 2014 led to a significant reduction in household purchases of sugary drinks, with a 12% decrease observed by the end of the year. The decline was more pronounced among lower-income households, highlighting the regressive nature of SSB consumption and the potential for taxation to address health disparities.^3^ Furthermore, projections suggest that taxes could decrease morbidity and mortality from diabetes and cardiovascular diseases, while also reducing healthcare costs.^3^

The UK’s Soft Drinks Industry Levy (SDIL) is a tax levied on soft drinks that include added sugar. It was announced in 2016 and implemented in April 2018 as an anti-obesity program to reduce sugar content in soft drink recipes. In addition, the tax aimed to change consumer behaviour by urging consumers to choose less expensive beverages with a reduced sugar content over more expensive drinks with higher sugar content has similarly demonstrated positive outcomes.^4^ The SDIL led to a 30% reduction in the volume of sugars sold per capita per day from soft drinks between 2015 and 2018, equivalent to a reduction of 4.6 grams per capita per day. Additionally, the tax incentivised manufacturers to reformulate products, resulting in a decrease in the sugar content of soft drinks and a shift towards lower-sugar alternatives.^4^ Together with Finland, Hungary, and Denmark, France was among the first European countries to introduce a tax on SSBs in 2012. France’s SSB tax initially applied a low, flat rate to all non-alcoholic drinks with added sugar or sweeteners, with its focus shifting over time from public health to budgetary concerns. In 2018, the tax was redesigned into a progressive, tiered system to encourage sugar reduction in products and promote healthier reformulation by manufacturers.^5^

In 2018, SA became the first country in Africa to introduce such a policy through the SSB tax, an excise tax on sugary beverages.^6^ Research conducted since the implementation of the SSB tax^4,7^ suggests modest reductions in SSB purchases. Still, there is limited understanding of public awareness and perceptions of the SSB tax, especially in smaller towns and rural communities. Here, socioeconomic realities, cultural practices, and access to affordable, healthy food may shape responses differently than in larger cities.

Nursing practitioners play a vital role in health education and promotion at the primary care level. As the first point of contact for many patients, particularly in rural areas, they are well-positioned to influence community understanding of healthy dietary practices. Their awareness of and attitudes toward the SSB tax are therefore critical, as they act as both providers of care and educators within their communities.

Despite the policy’s significance, there is currently limited documented evidence regarding the perceptions and awareness of the SSB tax among both consumers and frontline health workers in rural SA. Available literature focuses on national-level analyses or large urban communities, with limited insights from small, rural communities.^6,8–10^ This restricts understanding of how SSB taxation is perceived at the household and individual level, and whether it is regarded as a viable strategy for promoting healthier diets. Exploring these perceptions in Makhanda offers an opportunity to generate relevant evidence that informs understanding of consumer behaviours and perceptions shaped by socioeconomic constraints. In contrast, nursing practitioners provide insights from healthcare professionals who can influence community health education and advocate for policy compliance. This dual perspective enables a comprehensive understanding of knowledge, attitudes, and perceptions toward SSB taxation in a low socioeconomic status South African rural community.

Rather than depending solely on public awareness campaigns, the success of SSB taxation is shaped by broader policy networks, stakeholder influence, and governance dynamics that determine how the policy is framed, adopted, and implemented.^11^ For example, in Mexico, a civil society coalition led a media advocacy campaign before industry opposition, enabling proponents to shape public discourse in favour of the tax.^11,12^ The World Health Organisation (WHO) has similarly emphasised the importance of mobilising civil society and the medical community to counter industry resistance and promote public health messaging.^8^ One key factor in the successful adoption and impact of such fiscal measures is an effective public communication strategy—one that is timely, focused, and contextually relevant.

Understanding local perceptions of SSB taxation is crucial for optimising public health interventions. Findings from this study may guide strategies to enhance awareness, acceptance, and effectiveness of fiscal policies, thereby supporting healthier dietary practices and reducing the NCD burden in SA. Moreover, these insights can inform policymakers, health practitioners, and researchers seeking to implement context-sensitive nutrition interventions in similar rural communities.

However, important gaps remain, including limited community-level evidence on awareness and perceptions of the SSB tax, insufficient understanding of attitudes in low-socioeconomic settings, and minimal insight into nursing practitioners’ perspectives and the tax’s influence on SSB consumption behaviours. Considering these gaps, this study aimed to explore both the public and professional understanding of the SSB tax in a rural South African context. The research comprised two components. The first, a quantitative component, assessed awareness, beliefs, and self-reported changes in purchasing and consumption behaviour among adult clinic attendees. The second, a qualitative component, explored nursing practitioners’ awareness of the SSB tax, their perceptions of its effectiveness, and their views on their role in community education related to SSB consumption.

## Design and methods

This study employed a cross-sectional, multi-method design to explore and analyse the perspectives of adults and nursing practitioners regarding the SSB tax implemented in SA in April 2018 in Makhanda, Eastern Cape, SA. It was conducted at PHC facilities in Makhanda, Eastern Cape, from April 2023 to May 2023.

### Study population, sample selection, and size

The study was conducted in Makhanda, a town in South Africa’s Eastern Cape, situated within the predominantly rural Makana Local Municipality, and is characterised by a modest population size of 60 000, marked socio-economic inequality, high unemployment and poverty levels, and mixed urban–peri-urban geography, limited access to healthcare services, and a population reliant on low-cost food and beverage options.

A multi-stage sampling technique was used. The researcher used convenience sampling to select PHC facilities. Public healthcare facilities in Makhanda were included in the study due to their ease of access and logistical considerations. A total of seven PHC facilities were included in the study.

Participants for the quantitative component of the study were sampled using convenience sampling. The study’s inclusion criteria for the quantitative component were the following: (i) consenting adults aged 18 years and above and living in Makhanda, (ii) Able to converse in English, Afrikaans, and/or isiXhosa. A sample size of 196 participants was calculated to estimate a 15% proportion of participants expected to know about the SSB tax, assuming 5% precision and a 95% level of significance. Data collection with adult participants was concluded after recruiting 275 adults.

Sampling for the semi-structured interview was done purposively. This consisted of nursing practitioners employed at the respective PHC facilities who provide nutrition education to adults. The inclusion criteria for the qualitative component were the following: (i) Consenting professional and enrolled nurses employed at PHC facilities in Makhanda providing nutrition education to adults, (ii) Professional and enrolled nurses who have daily contact with adults at PHC facilities. Data saturation was determined through an iterative process of concurrent data collection and analysis. Codes and themes were continuously reviewed following each interview to assess the emergence of new information. By approximately the eighth interview, no substantially new themes were identified, with subsequent interviews (nine and ten) serving to confirm and refine the existing thematic framework rather than generate novel insights. Data collection was therefore concluded at ten interviews, as thematic redundancy had been achieved.

### Data collection

The data collection took place over two weeks, from 24 April 2023 to 05 of May 2023, at the identified PHC facilities in Makhanda, Eastern Cape, South Africa.

The quantitative data were collected from Monday to Friday between 8:00 am and 12:30 pm, as this was the busiest time for the PHC facilities with many prospective participants in the waiting areas. The researcher and two research assistants collected the data. On the days of data collection, adults in the waiting areas of the respective PHC facilities were approached and screened for inclusion in the study. Once screened, written informed consent was obtained in the participant’s language of choice. The participant received a copy of the consent form and was allocated a participant identification number. The interviewer-administered questionnaire took 15-20 minutes to complete, and participants were given a food parcel valued at R50 (3.21 USD) as a token of appreciation upon completion. The semi-structured interviews were conducted solely by the researcher. Prospective participants were screened according to their nursing qualifications, the frequency of patient contact, and whether they provided health education. Once screened for eligibility, written informed consent was obtained from each participant before data collection commenced. Each participant was allocated a unique identification number to ensure anonymity. The average duration of the interview was 41 minutes. After completing the interview, each nursing practitioner was given a Pick and Pay voucher to the value of R150 (9.35 USD) as a token of appreciation.

### Data collection tools

The interviewer-administered questionnaire was used to assess adults’ awareness, beliefs, perceptions, and practices regarding the SSB tax at PHC facilities. The questionnaire was developed by the researcher and was based on the study objectives. The questionnaire consisted of four sections: (i) demographic information, (ii) awareness, knowledge, and understanding, (iii) perceptions and beliefs of the SSB tax, and (iv) purchasing practices. Pre-set responses and yes/no options were used to assess awareness, knowledge, and understanding. Four-point Likert scales (strongly disagree, disagree, agree, strongly agree) were used to assess perceptions, beliefs, and practices. A four-point Likert scale (as used in the interviewer-administered questionnaire) is used in research to assess perceptions, beliefs, and practices because it forces participants to take a stance, avoiding a neutral or middle option. This design is particularly useful when researchers want to capture clear, directional opinions rather than indecisive or neutral responses. This approach aligns with the study objectives. Content validity was assessed by three experts in nutrition and the taxation of SSBs. Adaptations were made to the questionnaire based on their suggestions. The experts also examined the relevance of the questions and offered additional suggestions for including components the researcher had overlooked. The discussion guide for the semi-structured interviews consisted of 11 questions and was complemented by follow-up and probing questions. The discussion guide was developed by the researcher and is based on the study objectives. Content validity was assessed by three experts in nutrition and taxation regarding SSBs. All comments received were assessed, and adaptations were made accordingly.

### Pilot study

To assess face validity, pilot studies were conducted before the main data collection. For the adult questionnaire, a pilot study was conducted in March 2023 at Settlers Hospital, Makhanda, among 10 convenience-sampled adults attending the outpatient department. The pilot identified practical concerns, such as survey completion time, question clarity, and response types, and participants provided feedback on the questionnaire’s suitability and length. Based on these findings, the questionnaire was refined by clarifying misunderstood items and simplifying wordy questions.

A pilot study of semi-structured interviews with nursing practitioners was also conducted in March 2023, using a convenience sample of a practitioner from Settlers Hospital’s outpatient department. This pilot assessed test procedures, anticipated responses, and gathered feedback on the clarity and structure of the discussion guide. Following the pilot, the guide was refined by simplifying complex questions and translating them into Afrikaans to accommodate local language use. Data from both pilot studies were not included in the main study, but were essential for refining the instruments and ensuring smooth data collection.

## Quality assurance

### Training of research assistants

Before conducting the interviewer-administered questionnaires, the researcher and the two research assistants held a training session on using the standard operating procedure document during data collection.

### Reducing bias

For the semi-structured interviews, participants were not informed that the researcher was a registered dietitian, as this might lead them to believe they should provide answers a dietitian would want to hear.

### Potential selection biases

Although the study aimed to capture diverse perspectives, potential selection biases are inherent due to the sampling strategies and sample size. Adults were recruited using convenience sampling at PHC facilities, which may have led to over-representation of individuals who actively access healthcare services and under-representation of those who are less likely or unable to attend such facilities. Similarly, nursing practitioners were purposively sampled from specific hospitals and clinics, which may not fully reflect the views of practitioners in other healthcare settings across Makhanda. Furthermore, the relatively small sample size, while sufficient to reach thematic saturation, may limit the generalisability of findings and the detection of less common perspectives. Despite these limitations, careful efforts were made to include participants from diverse socioeconomic backgrounds, age groups, and levels of professional experience to maximise the diversity of insights captured.

### Ensuring trustworthiness

The researcher corroborated information from multiple individuals at the respective PHC facilities (obtained through semi-structured interviews and nonverbal expressions during the interviews) and described the methods in sufficient detail for someone replicating the study to obtain similar findings. This is with the expectation of variability inherent in qualitative studies.

## Ethical approval

This study was conducted according to the guidelines laid down in the Declaration of Helsinki, and all procedures involving human subjects were approved by the Health Research Ethics Committee of Stellenbosch University, Cape Town, South Africa (S22/01/014) and the Eastern Cape Department of Health via the National Health Research Database (EC_202209_002). Written informed consent was obtained from all subjects.

## Data analysis

**Figure 1.**
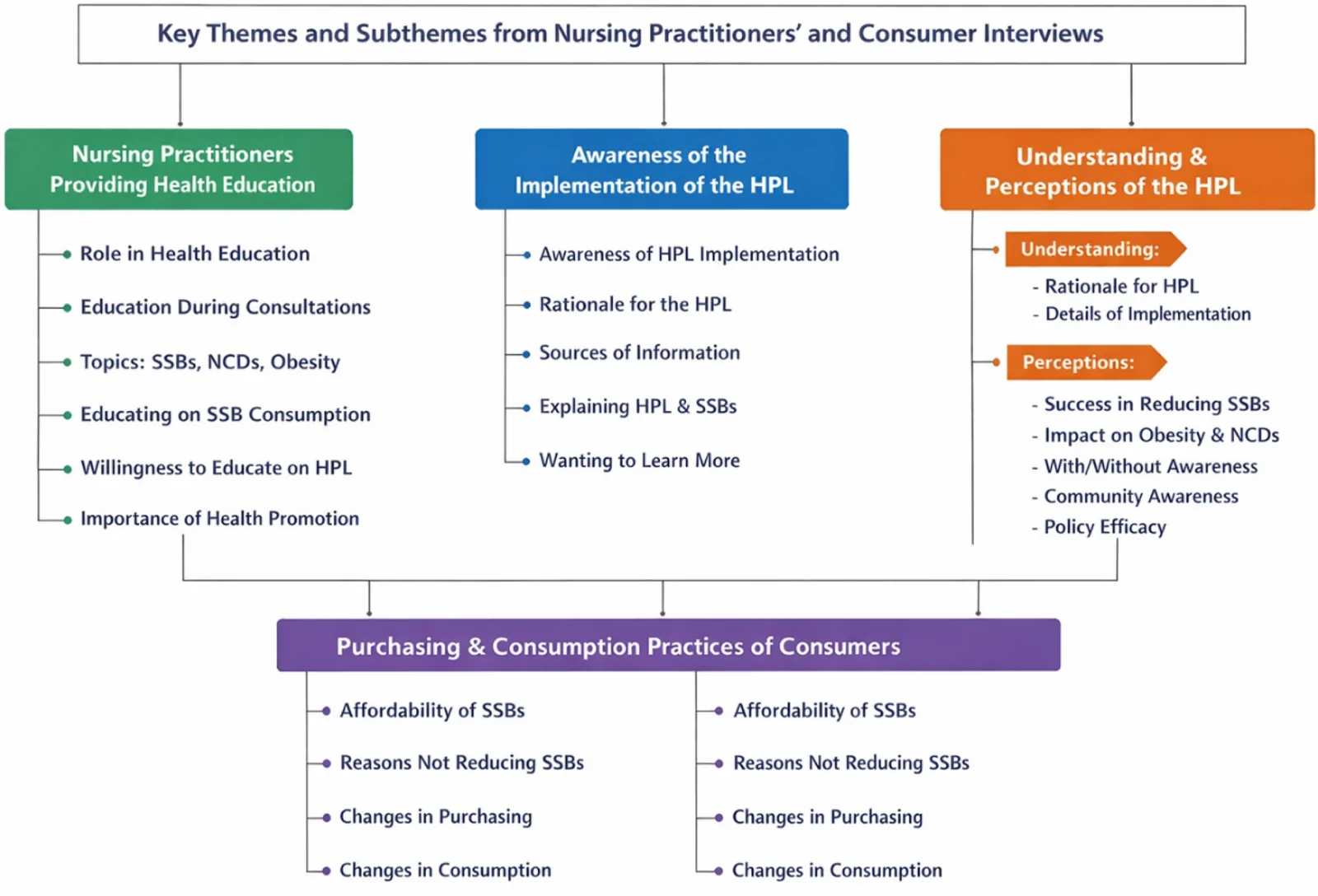
Key themes and sub-themes identified during qualitative data analysis.

Quantitative data from the interviewer-administered questionnaire were captured using Microsoft Excel 365. Study sample characteristics were summarized using descriptive statistics, and variable distributions were presented as frequency tables and/or histograms. The open-ended questions in the questionnaire were analysed by counting responses and grouping them into categories based on similarity. The semi-structured interviews were audiotaped and transcribed, and the transcripts were uploaded to ATLAS—ti 9. The coding process began deductively, using an initial set of codes based on the research questions. As new themes emerged, new codes were created inductively and added iteratively.^13^ Please see Figure 1 for the key themes and subthemes identified. The researcher was assisted by a trained data coder who performed quality control measures to ensure that the data were coded correctly. After analysing the quantitative and qualitative data, the two datasets were integrated, and their findings were compared during the discussion.

Missing data were examined for all variables. Where applicable, incomplete responses were addressed using pairwise deletion, and variables with notable missingness (≥5%) were flagged. Sensitivity analyses were conducted to assess the potential impact of missing data on the study findings, ensuring the transparency and robustness of the results.

## Results

The results from the interviewer-administered questionnaire and the semi-structured interview are presented, as per the study’s objectives, and integrated into the discussion. The key findings from the semi-structured interviews, identified through pre-existing and emerging themes, are reported below.

### Demographic characteristics

A total of 275 interviewer-administered questionnaires were completed, of which 62% (n = 170) were females and 38% (n = 105) males, with a mean age of 42 ± 13.9 years, ranging from 18 to 82 years. Most participants (54%, n = 149) had completed secondary education, 29% (n = 80) had completed only primary schooling, and 17% (n = 47) had some form of tertiary education. Regarding employment, 61% (n = 168) were unemployed, 24% (n = 66) were employed, and 5% (n = 14) were students or engaged in informal work. With the majority of the participants (19%, n = 52) attending the NG Dlukulu Clinic. Table 1 presents the demographic profile of adult participants.

**Table 1.** The demographic profile of adult participants (n=275).

*Age (y)	n (%)	Gender	n (%)
18-29	53 (19%)	Female	170 (62%)
30-39	71 (25%)	Male	105 (38%)
40-49	76 (28%)	Ethnicity	​
50-59	34 (13%)	Black	225 (82%)
≥60	41 (15%)	Coloured	50 (18%)
Home Language		White	0 (0%)
Afrikaans	50 (18%)	Other	0 (0%)
isiXhosa	223 (81%)	I don’t want to say	0 (0%)
English	0 (0%)	Level of Education	​
Other	2 (1%)	Primary	32 (12%)
*Total household income after deductions		Secondary	177 (64%)
No income	59 (22%)	Matriculated	55 (20%)
R1–R4 800	172 (63%)	Tertiary	11 (4%)
R4 801–R9 600	35 (13%)	*Employment Status	​
R9 601–R19 600	0 (0%)	Full-time	42 (15%)
R19 601 or more	1 (0%)	Part-time	44 (16%)
I do not know	4 (1%)	Student	9 (3%)
I do not want to tell	1 (0%)	Retired	21 (8%)
​		Unemployed (actively seeking employment)	132 (48%)
​		Unemployed (not actively seeking employment)	27 (10%)
PHC			
Raglan Road Clinic	30 (11%)	Settlers Day Clinic	42 (15%)
Middle Terrace Clinic	50 (18%)	Anglo African Clinic	31 (11%)
NG Dlukulu Clinic	53 (19%)	V Shumane Clinic	24 (9%)
Joza Clinic	45 (16%)	​	​
NG Dlukulu Clinic	53 (19%)	V Shumane Clinic	24 (9%)
Joza Clinic	45 (16%)	​	​

y, year; PHC, primary health care facility.

*cut-off values according to South African census data (Statistics South Africa Community Survey, 2016).

The semi-structured interview participants comprised ten nursing practitioners, of whom nine were female, and one was male, representing all seven PHC facilities.

### Awareness, beliefs, and understanding of the SSB tax

Overall, awareness of the SSB tax was extremely low. Most adult participants (96%, n = 264) were unaware of the SSB tax, and tax 89% (n = 245) did not know what the term SSBs meant. Only 11 (4%) participants had heard of the tax, and of these, just four correctly identified its introduction in 2018 and explained it in their own words as “drinks with added sugar”. If participants were unaware of the SSB tax and the term SSB, the interviewer briefly defined the SSB tax to allow them to continue answering questions about it. Table 2 summarises participants’ awareness and perceptions of the HPL.

**Table 2.** Adult participants’ awareness and perceptions of the SSB tax (n=275).

Question	Yes	No
	n (%)	n (%)
Do you know what the term SSBs mean?	30 (11%)	245 (89%)
Are you aware of the SSB tax, also known as the HPL, which was implemented in SA?	11 (4%)	264 (96%)
Do you think the taste of SSBs are different after the implementation of the sugar tax?	151 (55%)	124 (45%)

SSBs, sugar-sweetened beverages; HPL, health promotion levy; SA, South Africa.

Interestingly, more than half of the participants perceived that SSBs were ‘not as sweet as before,’ suggesting industry reformulation in response to the SSB tax. This implies that even though consumers report no change in the quantity of SSBs they purchase, the sugar content of those beverages may have dropped, leading to a reduction in sugar intake independent of consumers’ purchasing behaviour. Reasons cited included: “not as sweet as it used to be” (n = 52; 19%), “less acid” (n = 39; 14%), and “an aftertaste” (n = 20; 7%).

**Figure 2.**
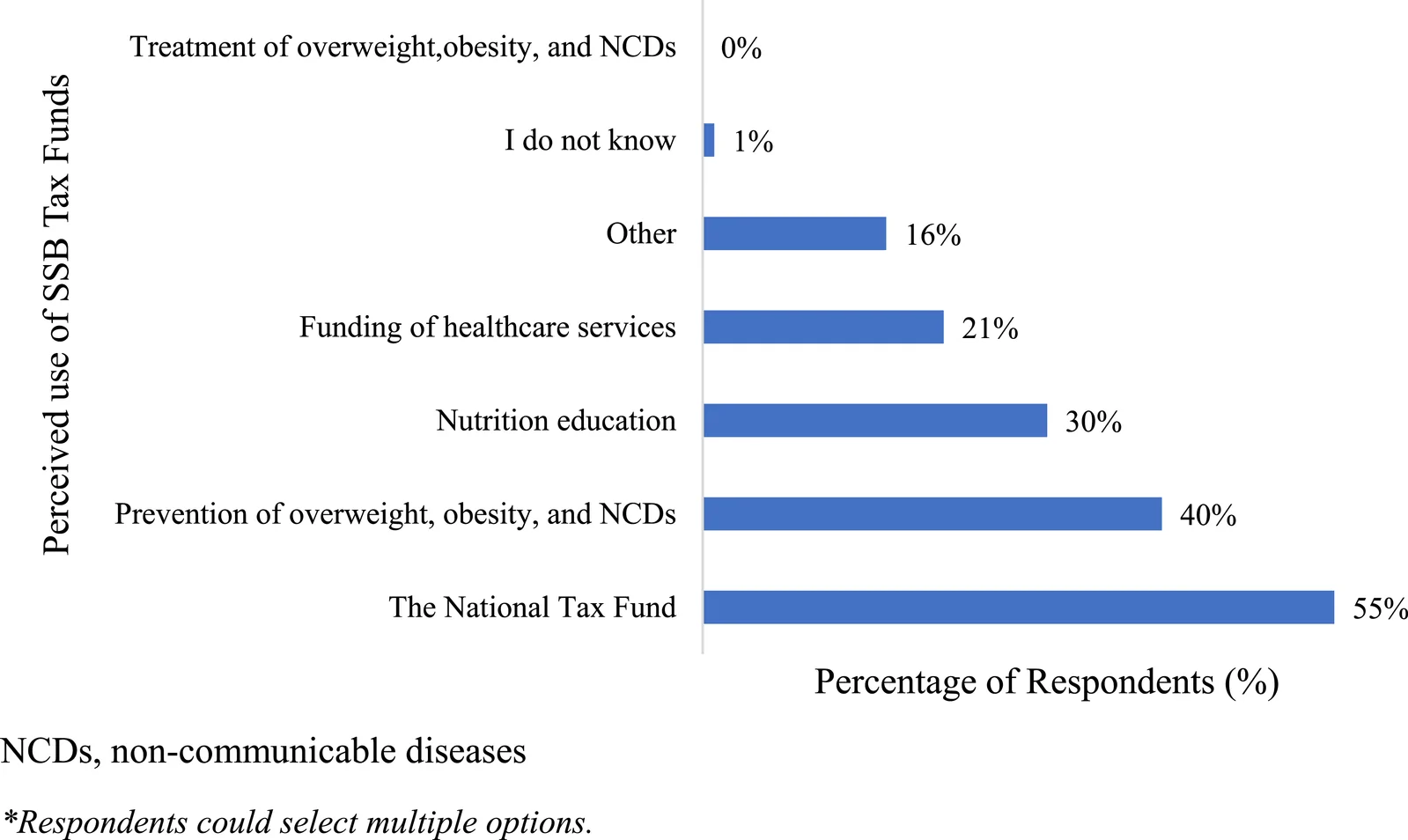
Adult participants’ perceptions of what money collected from the HPL is used for (n=275).

Figure 2 summarises adult participants’ perceptions of how the SSB tax revenue should be used. Most participants felt it should fund healthcare services and improve PHC facilities in their community.

From the nursing practitioners’ perspective, limited knowledge was also evident. Several participants recalled hearing about the tax in the media but admitted they had not paid attention to the details.

“Okay, so I actually do not know anything about the health promotion levy. I think I have heard; you know, when you listen to something in the background, and that is what I did with the budget speech.” – Female, nursing practitioner 1

Nursing practitioners mentioned that the consumer’s lack of knowledge regarding their SSB intake, the effects of that intake on their health, and low health literacy pose a challenge to the success of the SSB tax. They emphasised that without their own training and knowledge of SSB tax, they cannot educate communities effectively, and agreed that awareness among practitioners is essential to the policy’s impact.

### Perceived role of nursing practitioners in educating adults

There was a strong consensus that health education forms an integral part of the job description of a nursing practitioner. It was noted that health education is an important part of a consultation session and that opportunities to educate patients should not be missed, especially in communities with low health literacy. Nursing practitioners stressed that they are the most frequent point of contact patients have with the health care system; therefore, health education is important.

“I think it is part of the scope of a nursing practitioner to provide education to the patient that they see. We need to ensure, as health care professionals, that accurate information reaches our communities, because health literacy levels are very low. So, it is very easy for someone to have a misconception about something.” Male nursing practitioner

Nursing practitioners agreed that SSB education is important, but admitted they seldom address it unless patients have diabetes or ask directly. Barriers included limited knowledge of SSBs, their health effects, and healthier alternatives. They noted that training on the SSB taxation policy would increase their confidence to counsel patients.

“I think once we get a little bit of training or some form of a pamphlet or material to back whatever you are saying, just to give you that confidence, because I mean, nutrition is not our specialty.” Female nursing practitioner 3

Nursing practitioners play an important role in creating awareness and educating patients, and by ensuring that they themselves are educated on the SSB tax, they can educate and raise awareness amongst their patients about the dangers of overweight, obesity, NCDs, and SSB intake.

### Perceptions on the effectiveness of the SSB tax

Adult participants expressed mixed views on the SSB tax. While 77% (n = 212) supported it, many saw it as a government money-making scheme. Opinions were split on whether it would reduce obesity (48%, n = 132 agreed vs 50%, n = 138 disagreed) or harm the economy through job losses (52%, n = 143 agreed vs 47%, n = 129 disagreed). Most believe it would not reduce consumption, with some noting that people “love these drinks” and remain unaware of their health risks.

There was a strong consensus among the nursing practitioners that the SSB tax is an effective measure by the South African government to decrease the consumption of SSBs and address the rising epidemic of overweight, obesity, and NCDs. Nursing practitioners agreed that a population-wide approach will ensure greater gains for the population and that the effectiveness of the SSB tax will rely on multi-sectoral collaboration, which needs to include commitment from beverage companies to reformulate their products and from the government to support and drive consumer awareness programs.

“I think it is a good thing because our people do not take responsibility for their health. They (people) always rely on the government, and I think that is something good because they are not going to, out of their own, be like okay, so I need to have less SSBs.” Female nursing practitioner 4

It was clear that large-scale media and health promotion campaigns are key drivers of the SSB tax’s success. Nursing practitioners mentioned that if the South African government treated overweight, obesity, and NCDs with the same urgency as HIV and the Coronavirus, and if media campaigns were as successful and internalised by the South African population as those for HIV and the Coronavirus, it would be successful.

### Self-reported and perceived changes in SSB purchasing and consumption

**Figure 3.**
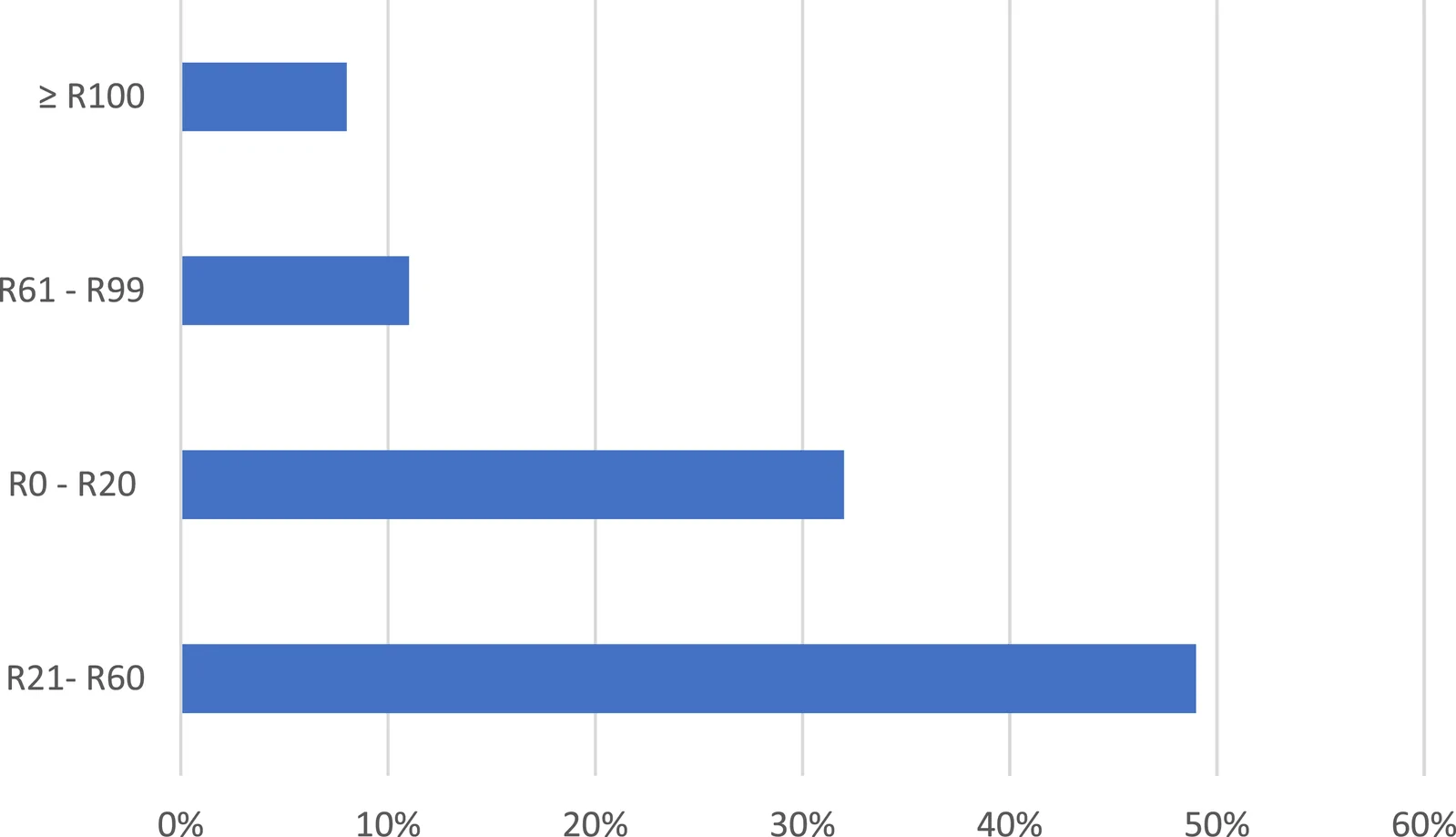
Adult participants’ responses on weekly expenditure on SSBs (n=275).

Adult participants reported that their consumption and purchasing practices have not been affected by the SSB tax, as 95% (n = 261) reported consuming SSBs. When asked whether they ever buy SSBs, 96% (n = 264) of the adult participants responded “yes” and reported making the purchasing decision themselves. When asked about the frequency of purchasing SSBs, the majority of adult participants reported buying them weekly, and most purchases were made at a spaza shop. Most adult participants reported that it takes them less than five minutes to decide to buy SSBs when they are in a shop, café, or spaza shop. Figure 3 summarises the responses for weekly SSB purchases. Most adult participants reported that they had not stopped buying SSBs because of the SSB tax, and when asked whether the SSB tax decreased their SSB intake, 91% (n = 250) strongly disagreed/disagreed.

Nursing practitioners felt that the SSB tax has not changed consumer behaviour because the price increase was marginal and SSBs remain affordable, even for low-income households. They noted that consumption is driven as much by habit as by taste, and that South Africans are often reluctant to change dietary behaviour despite health risks. However, they agreed that a substantial price rise would likely reduce purchases.

“I think they (individuals) are still buying the same amount because I mean the prices have stayed the same. There's nothing that would have made them buy less. Yes, there is an HPL that no one is aware of that was implemented, but did the prices change? No, it did not change a lot. I mean, we thought the prices increased because of increases in prices with the economy as it is.” Female nursing practitioner 4

Nursing practitioners agreed that reducing the population’s SSB intake is the appropriate approach and that consumer awareness programs are integral to this strategy. They agreed that the South African government needs to increase the prices more drastically to see change.

## Discussion

This study provides a baseline assessment of adult and nursing practitioners’ perceptions of the SSB tax in a low socio-economic status community in Makhanda, Eastern Cape, SA. As the first study in this context, it highlights critical gaps in awareness and understanding of this key public health policy aimed at reducing SSB consumption. The findings underscore that effective policy implementation requires not only legislation but also comprehensive communication, health education, and multi-sectoral collaboration.

Despite national media campaigns both against and in support of the SSB tax, both the quantitative data (low reported awareness levels) and qualitative accounts (participants expressing unfamiliarity with the policy) indicate that participants were largely unaware of its implementation. This aligns with findings by Bosire et al.,^9^ who reported limited awareness of SSB taxation among urban South Africans, yet contrasts with Koen et al.,^10^ where Cape Town consumers demonstrated higher awareness. Differences likely reflect disparities in education and income, highlighting that socio-economic and literacy factors influence the reach of policy. Effective implementation must therefore include strategies tailored to low-income communities to ensure equitable awareness and understanding.

Nursing practitioners, as frontline public health providers, play a critical role in providing health education and information to the community.^14^ Qualitative findings showed that while most reported delivering general health information, quantitative insights revealed limited awareness of the SSB tax itself and minimal engagement in educating patients about SSB consumption. This professional knowledge gap helps explain the low public awareness observed, as it suggests that limited provider knowledge constrains patient education at PHC facilities in Makhanda. Consistent with Essman et al.,^14^ these findings suggest that the enactment of a tax alone is insufficient to influence public perceptions and behaviours; structured communication and health education initiatives are essential to explain the purpose and benefits of the policy.

A notable finding was that adult participants expressed conditional support for the SSB tax. Qualitatively, levels of support appeared relatively favourable; however, quantitative data contextualised this support by revealing scepticism, with participants often framing the tax as a government revenue-generating scheme. This perception reflects broader societal concerns about corruption and declining tax morale in South Africa and aligns with international evidence indicating SSB taxes are sometimes viewed primarily as fiscal instruments rather than public health interventions. Social desirability bias may also explain the discrepancy between reported support (qualitative) and sceptical narratives (quantitative), as respondents may feel compelled to endorse health-promoting policies despite personal reservations or behaviours.

Most adults reported that the SSB tax had little impact on their purchasing or consumption behaviours, a finding supported quantitatively and elaborated qualitatively through explanations of habit, taste preference, and the relatively small price increase. Nursing practitioners similarly perceived limited behavioural change among their patients, reinforcing this pattern across both datasets. These findings are consistent with Bosire et al.^9^ and Ortega-Avila et al.,^15^ who highlighted that SSB consumption is shaped by multiple factors beyond price, including habit, addiction, availability, and advertising. However, they contrast with findings from Koen et al.^10^ and Stacey et al.,^16^ who reported reductions in SSB purchases following tax implementation, particularly among populations with higher levels of education. This divergence further highlights the role of socio-economic context in shaping the effectiveness of fiscal health policies.

The study also revealed a divergence in perceptions of the SSB tax’s effectiveness in reducing obesity. Qualitative results indicated stronger support, while quantitative findings showed that adult participants expressed scepticism regarding its impact. This discrepancy likely reflects differences in educational background and professional exposure to public health principles. Consistent with Murukutla et al.,^17^ mass media campaigns can improve knowledge of SSB-related health risks and build public support for taxation. Digitising and expanding the scope of health education programs, as recommended by Rizvi,^7^ may further strengthen interventions by providing accessible, evidence-based information to communities with low literacy levels.

The limited effect of the SSB tax on purchasing behaviours observed in both datasets may also reflect the marginal price increases experienced by low-income consumers. Manyema et al.^18^ projected that a 20% SSB tax could significantly reduce obesity prevalence, suggesting that larger fiscal measures may be needed to achieve a substantial public health impact. However, the qualitative findings emphasise that taxation alone is unlikely to overcome entrenched consumption habits, reinforcing the need for complementary strategies such as health education, environmental modifications, and supportive policy measures alongside fiscal interventions.

These integrated findings have important implications for broader policy implementation. Evidence from both the quantitative and qualitative components indicates that successful public health taxation requires not only legislative action but also active dissemination, professional training, community engagement, and multi-sectoral collaboration. Nursing practitioners specifically emphasised the need for large-scale media campaigns. They coordinated health promotion as key enablers of the SSB tax’s effectiveness,^17^ aligning with international evidence that such taxes are most effective when combined with public education, accessible healthier alternatives, and supportive interventions.

In summary, this study demonstrates that while the SSB tax is broadly supported in principle, public awareness and understanding remain limited in low socio-economic settings. The limited awareness of the SSB tax observed in this study may, in part, be attributed to its design and implementation mechanism. As an excise tax applied at the manufacturer level, the SSB tax is incorporated into the final retail price of sugar-sweetened beverages rather than presented as a visible or itemised charge at the point of purchase. Consequently, consumers may not directly perceive the tax, reducing its salience and limiting its potential to influence awareness and behaviour.

This lack of visibility may explain why participants did not associate changes in product pricing with the SSB tax or recognise it as a public health intervention. These findings suggest that, in addition to fiscal measures, greater emphasis should be placed on improving the policy’s transparency and communication. Strategies such as enhanced public awareness campaigns, clearer product labelling, and point-of-sale messaging may increase consumer awareness and strengthen the overall effectiveness of the SSB tax. Perceptions of government motives and habitual consumption patterns further limit the policy’s immediate behavioural impact. Addressing these gaps requires comprehensive, context-specific communication strategies, professional training for healthcare providers, and community-targeted educational interventions. These strategies are critical to increase awareness, acceptability, and behaviour change, ultimately enhancing the SSB tax’s contribution to reducing SSB consumption, obesity, and NCDs in SA.

## Conclusion

Despite the overall lack of awareness of HPL among adult participants and nursing practitioners, the study highlights broad support for a population-wide strategy to reduce SSB consumption, lower sugar intake, and curb NCDs in SA. Nursing practitioners emphasised that the effectiveness of the SSB policy will rely on multi-sectoral collaboration, requiring commitment from both the beverage industry to reformulate its products and the government to lead consumer awareness initiatives, while ensuring transparency in the use of the revenue collected.

This study is the first SA to explore the awareness, beliefs, and perceptions of both recipients and providers of health education regarding SSB taxation in a rural, low-socioeconomic setting. These findings provide novel insights into how rural communities understand and respond to health policy intervention and highlight the need for targeted education, media campaigns, and integrated public health strategies to maximise the policy’s impact. By capturing the perspectives of both recipients and providers of health education, this std informs policy and program strategies aimed at reducing sugar intake in similar low-income settings.

## Recommendations

Based on the findings of this study, several measures are recommended to improve the awareness, understanding, and effectiveness of the SSB tax in SA, particularly in low-socioeconomic communities. Education and awareness campaigns should be contextually appropriate and specific, including television or radio adverts, billboards in high-traffic areas, and print media, such as pamphlets distributed at clinics and schools. The SSB tax policy should be incorporated into the school curriculum, particularly in subjects such as Life Orientation, from primary school. New avenues for spreading the message, such as social media platforms, should be explored, leveraging content creators and influencers on Instagram, TikTok, and Facebook to reach large audiences, particularly youth and young adults. Interactive campaigns, such as community workshops, school competitions, and mobile phone messaging services (SMS/WhatsApp campaigns), can reinforce key messages and encourage engagement. To ensure cohesive messaging and maximise the impact of interventions, government, beverage industry stakeholders, health professionals, and community leaders must collaborate in the design and implementation of these campaigns.

Further evidence-based research is needed in several areas related to the SSB tax. Studies should focus on awareness and understanding of tax, purchasing, and consumption practices before and after its implementation in low socio-economic communities, as well as on the perspectives of integrated nutrition. Program managers at the district, provincial, and national levels.

This study provides baseline data; similar studies should be conducted in other provinces to capture regional differences, and follow-up studies should assess changes in awareness, perceptions, and behaviours over time to evaluate the effectiveness of education campaigns and policy measures. Strengthening health education programs at PHC facilities is also critical. Nursing practitioners must be knowledgeable about the SSB tax to effectively educate community members, address gaps in awareness, and promote informed behaviour change.

By implementing these recommendations, policymakers and public health practitioners can improve public knowledge, foster positive attitudes, and enhance the overall effectiveness of the SSB tax in reducing sugar intake and the burden of NCDs in SA.

## Limitations

This study has several limitations that should be considered when interpreting the findings. First, the use of convenience sampling for both PHC facilities and participants limits the generalisability of the results. While this approach was practical given the study context and resources, it may have introduced selection bias, as participants who were more accessible or willing to participate might differ systematically from those who were not included. Future studies using probability sampling or stratified approaches across multiple regions would strengthen the representativeness of findings.

Second, the study relied on self-reported data regarding awareness, perceptions, and behaviours. Although self-reported data is commonly used in public health research, it is subject to social desirability and recall biases. In this study, participants may have provided responses they perceived as favourable to the health researcher conducting the interviews, particularly when discussing their support for the SSB tax or consumption practices. This could have led to an overestimation of awareness or positive attitudes and an underestimation of SSB consumption.

Third, the cross-sectional design of the study limits the ability to assess temporal relationships or the causal impact of the SSB tax on purchasing and consumption behaviours. Observed associations represent a single point in time and cannot account for changes before or after policy implementation. Longitudinal or repeated measures designs would provide stronger evidence of behavioural and perceptual changes over time.

Lastly, the geographic scope of the study was limited to a single town, Makhanda, in the Eastern Cape. The finding may not be generalisable to other regions of SA, which differ in socio-economic status, educational attainment, and access to healthcare services. Conducting similar studies across diverse provinces would help to capture regional variations in awareness, attitudes, and behaviour related to the SSB tax.

In summary, while the study provides valuable baseline data on awareness, perceptions, and health education practices regarding the SSB tax, these limitations highlight the need for cautious interpretation and indicate avenues for more rigorous, generalisable, and longitudinal research in the future.
